# Non-syndromic Bilateral Supernumerary Teeth in the Primary and Secondary Dentition: A Rare Case Report

**DOI:** 10.7759/cureus.55385

**Published:** 2024-03-02

**Authors:** Maha Tbeishat, Araam M Odeibat, Ala' Ersheidat, Amira Mahasneh

**Affiliations:** 1 Dentistry, Princess Basma Teaching Hospital, Jordanian Ministry of Health, Irbid, JOR; 2 Medicine, Yarmouk University, Irbid, JOR; 3 Dentistry, Jordanian Royal Medical Services, Amman, JOR

**Keywords:** supplemental lateral incisors, radiographic imaging, secondary dentition, non-syndromic, primary dentition, supernumerary teeth

## Abstract

Supernumerary teeth are presented in any region of the dental arches as additional teeth to the standard set of teeth and may present in either the primary or secondary dentition. They can be found as single or multiple teeth on one or both sides of the dental arches, with a preference for the premaxilla. The supernumerary teeth might cause aesthetic and/or functional problems, mainly if situated in the maxillary anterior region. Multiple supernumerary teeth are often related to specific conditions or in syndromic patients, i.e., cleft palate, cleft lip, cleidocranial dysplasia, and Gardner's syndrome. This report presents a case of an eight-year-old female patient with non-syndromic bilateral supernumerary teeth in the primary and secondary dentition. The patient is medically fit, and her family history was non-contributory.

## Introduction

Supernumerary teeth are presented in any region of the dental arches as additional teeth to the standard set of teeth and may present in either the primary or secondary dentition.

The prevalence of supernumerary teeth is 0.3-0.6% in primary dentition, which is five times less than that in secondary dentition [1]; other studies reported the prevalence of supernumerary teeth for secondary dentition to be between 0.5% and 5.3% [2]. Males are more commonly affected with supernumerary teeth than females, with a reported ratio of 2:1. However, there is no significant difference in the gender distribution for primary dentition [3].

Supernumerary teeth can be found as single or multiple teeth on one or both sides of the dental arches, with a preference for the premaxilla. Furthermore, a classification can be made based on their location and morphology [4].

If a supernumerary tooth is situated in the maxillary anterior region, it can cause aesthetic and/or functional problems. In addition, supernumerary teeth in the primary dentition are usually unnoticed due to their normal shape appearance and their eruption mostly in a proper arch alignment [5].

The incidence of supernumerary teeth in both dentitions of the same child has been reported. It occurs in one-third of the cases [6]; it frequently involves supplemental instead of rudimentary forms and lateral rather than central incisors [1].

The etiology of hyperdontia is still not completely understood. Several theories suggest its occurrence, and the most accepted one is the hyperactivity theory, which indicates that supernumeraries are formed because of local, independent, conditioned increased activity of dental lamina [7].

The presence of supernumerary teeth may be a part of developmental disorders. The most common syndromes that show a significant incidence of multiple supernumerary teeth are cleft lip and palate [8].

The presence of supernumerary teeth might be responsible for alteration in the eruption of permanent dentition (26-52%), displacement or rotation of permanent teeth (28-63%), crowding, diastema, dilaceration or abnormalities in the root development of permanent teeth, cysts (4-9%), or eruption into the nasal cavity [9]. Early recognition and interceptive intervention are important for permanent dentition.

Cone beam computed tomography (CBCT) shows the intra-bony location, angulation, and shape of impacted supernumeraries, the distance from adjacent roots, and the distances between the impacted teeth and cortical bone [10]. Radiograph findings should be complemented with clinical findings to ensure proper diagnosis [11].

## Case presentation

An eight-year-old female patient attended our outpatient orthodontic clinic at Princess Basma Teaching Hospital in February 2022. The patient presented with non-syndromic bilateral supernumerary teeth in both the primary and permanent dentition and macrodontia of permanent central incisors, with a chief complaint of unerupted permanent central incisors. The patient is medically fit, and her family history was non-contributory.

**Figure 1:**
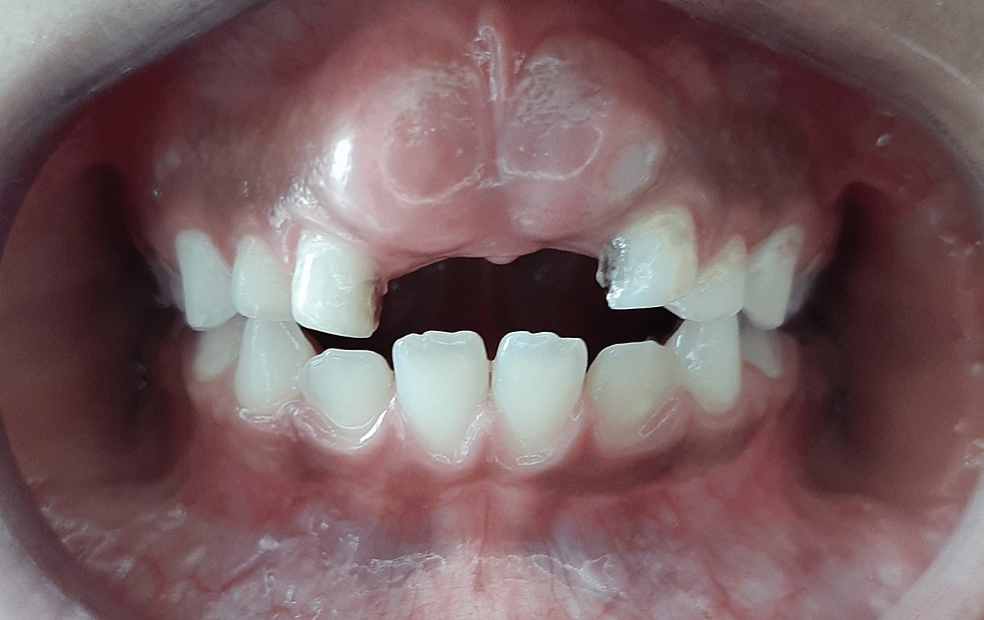
Intraoral photograph showing the bulge of unerupted secondary central incisors.

Intraoral examination showed missing right and left primary central incisors. The bulge of unerupted permanent central incisors is noticeable (Figure 1), with insufficient space in the line of the arch for both centrals to erupt. Two primary lateral incisors are on each side (supplemental tooth beside the primary lateral incisor).

**Figure 2:**
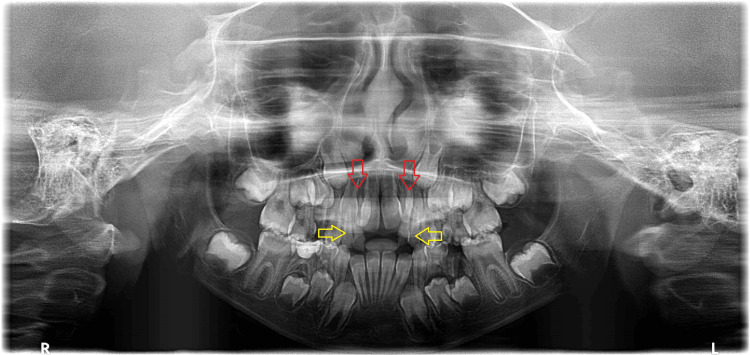
Panoramic radiograph. The yellow arrows point to the supernumeraries in the primary dentition, and the red arrows point to the supernumeraries in the secondary dentition.

Intraoral panoramic radiograph revealed the presence of two impacted supernumeraries (one on each side) beside the permanent lateral incisors (Figure 2) in addition to the supplemental teeth in the primary dentition.

**Figure 3:**
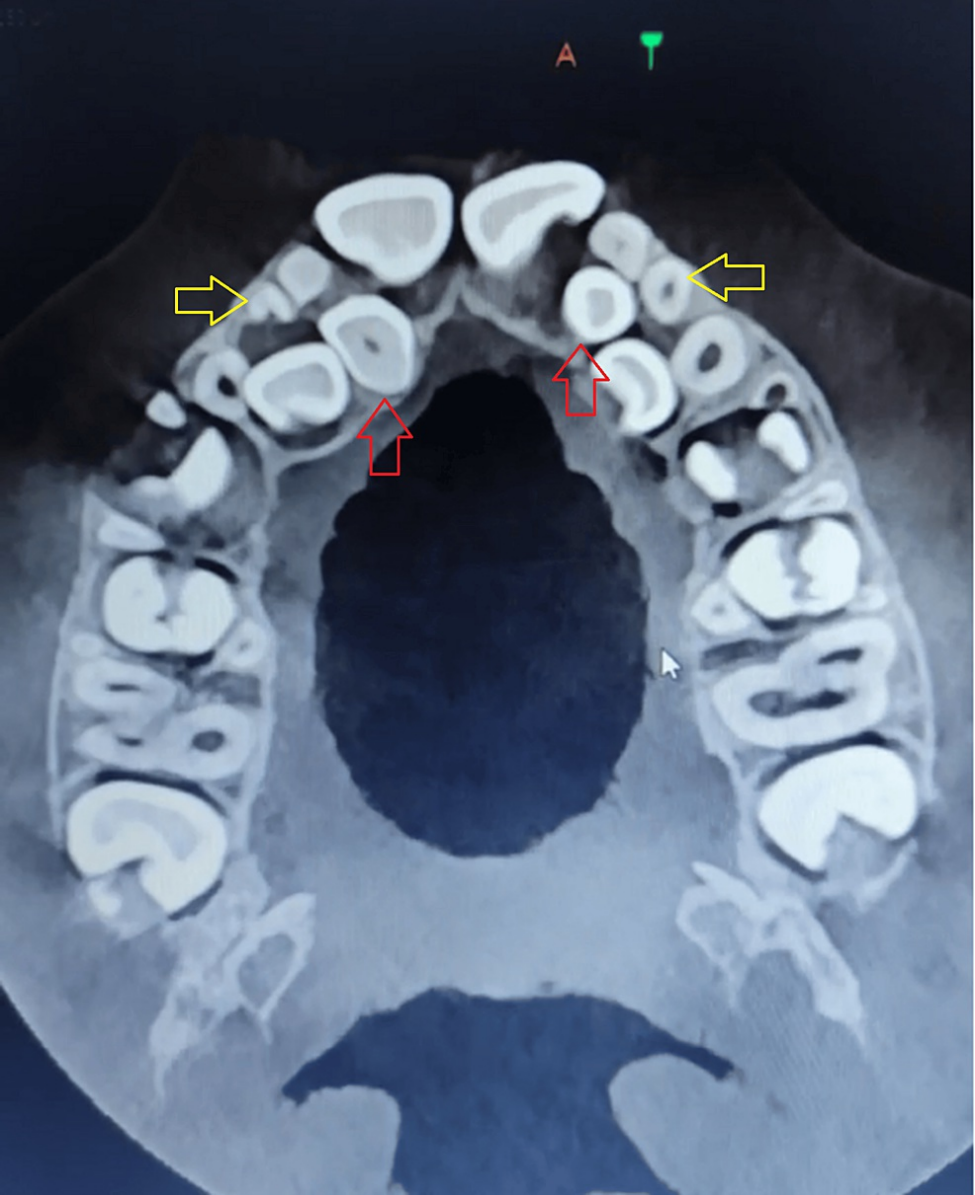
CBCT transverse section of the maxilla. The yellow arrows point to supernumeraries in the primary dentition, and the red arrows point to supernumeraries in the secondary dentition.

CBCT was taken for better diagnosis and treatment planning (Figure 3).

The patient was referred for bilateral extraction of the upper primary lateral incisors and kept under observation till the eruption of permanent central incisors.

After five months, the permanent central incisors were partially erupted, and the patient was referred to extract the remaining B’s.

**Figure 4:**
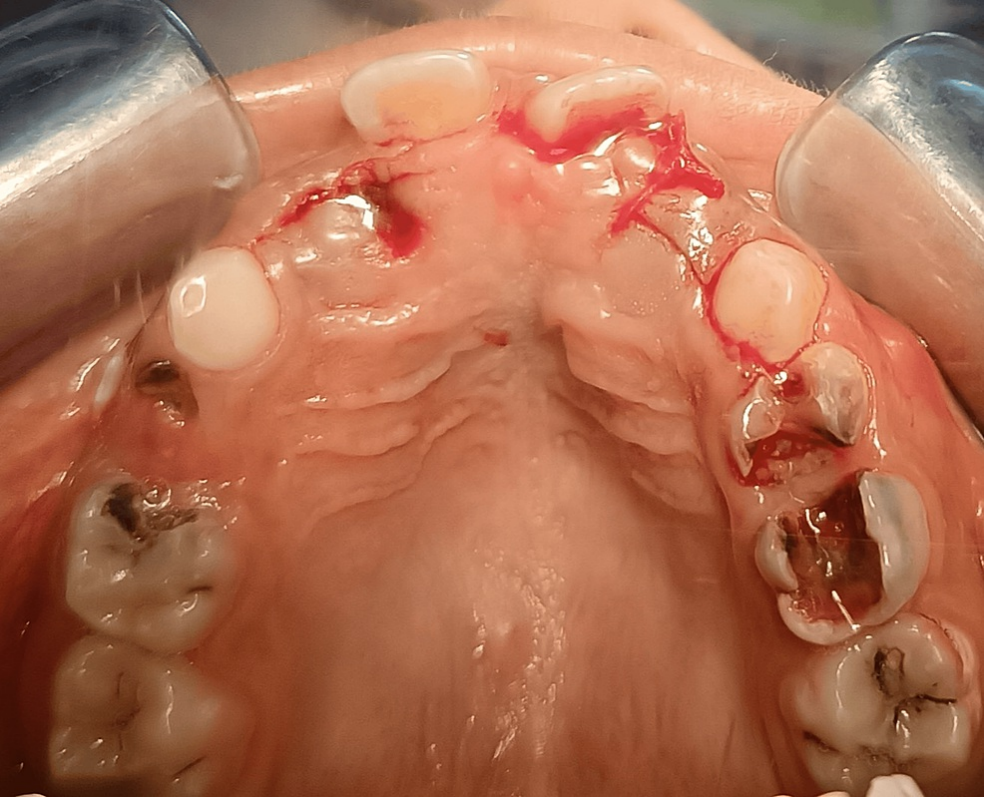
Occlusal view immediately after surgical extraction of the secondary supernumerary teeth

Following the remaining B’s extraction, the patient was referred to surgically extract the supplemental lateral incisors because they prevented lateral incisors from erupting into normal occlusion. A palatal flap was raised for that purpose (Figure 4).

**Figure 5:**
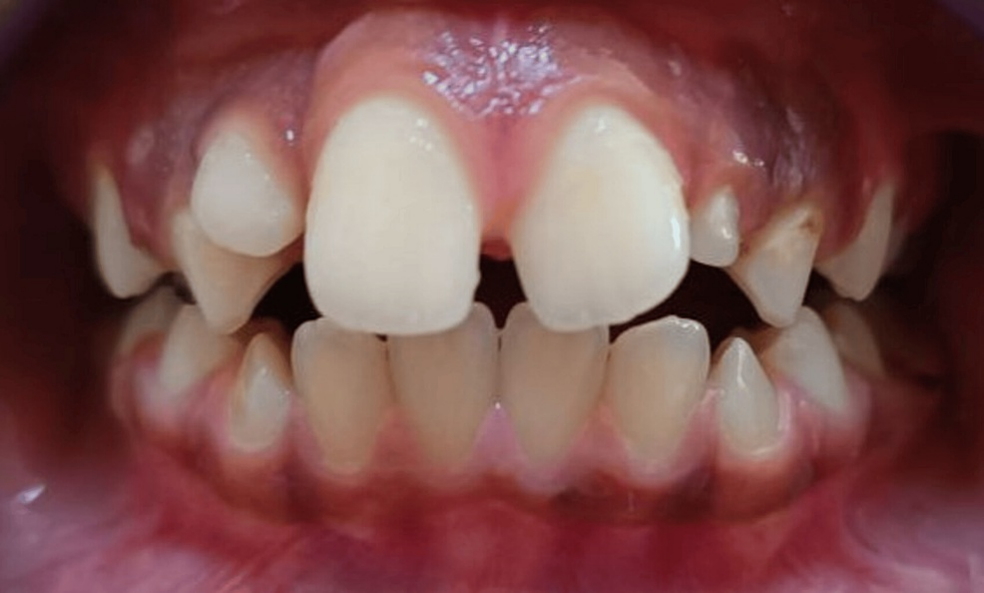
Intraoral frontal photograph showing the erupting permanent Incisors.

The patient was seen periodically every two months to monitor the spontaneous eruption of lateral incisors (Figure 5).

**Figure 6:**
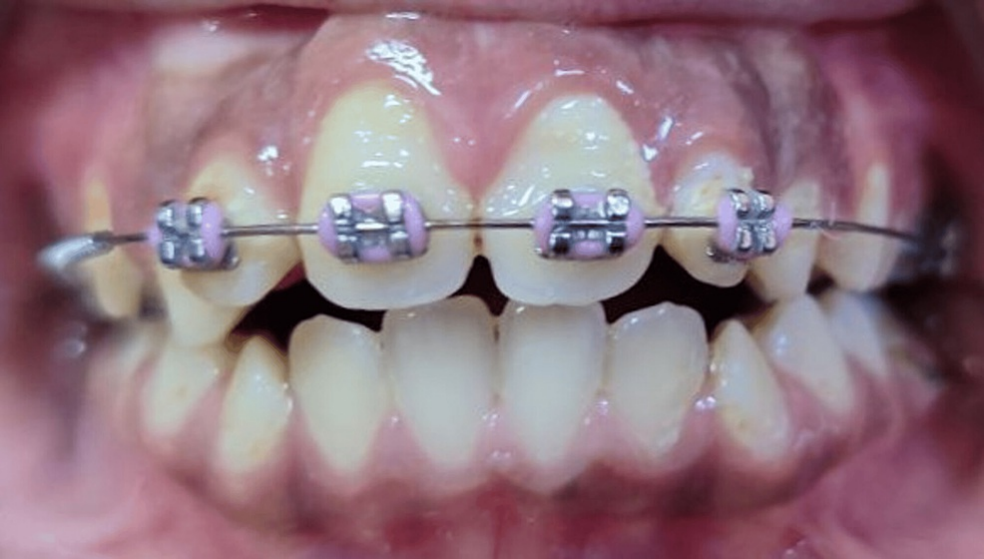
Intraoral frontal photograph showing the interceptive orthodontic treatment using a 2x4 partial fixed appliance

After the eruption of lateral incisors into normal occlusion, interceptive orthodontic treatment using a 2x4 partial fixed appliance to align the teeth was performed (Figure 6).

**Figure 7:**
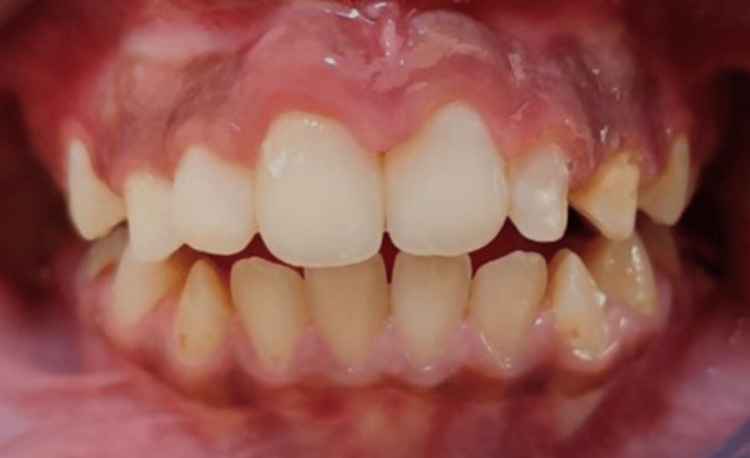
Intraoral frontal photograph after removing the 2x4 appliance

Nine months later, at the end of the 2x4 partial fixed interceptive orthodontic treatment (Figure 7), a comprehensive orthodontic treatment was recommended for the patient after the completion of the eruption of the secondary dentition.

## Discussion

Supernumerary teeth may present in the primary and permanent dentition. Moreover, supernumerary teeth can be found in syndromic patients, and rare incidents occur in non-syndromic individuals. Several studies reported that 76-86% of non-syndromic cases involve only a single supernumerary tooth and that 12-23% of cases present more than one supernumerary tooth [12]. Other studies reported the prevalence of supernumerary teeth as 0.2-3% in the primary and permanent dentition [1,13]. In addition, supernumerary teeth in the permanent dentition are more common than in the primary dentition; patients with supernumeraries in primary dentitions have a 30-50% chance of supernumeraries found in their primary dentition [14,15]. In the literature, a few cases of non-syndromic or systemically related supernumerary teeth were reported [16-19].

There have been many attempts to explain the etiology behind supernumerary teeth; however, the dental lamina hyperactivity theory is the most accepted one. Genetic factors and inheritance patterns might also cause supernumerary teeth [20]. In such cases, treatment varies according to the underlying malocclusion. Clinical and radiographic identification of supernumerary teeth is crucial for treatment planning.

In our case report of an eight-year-old female with bilateral supernumerary teeth in both the primary and permanent dentition and macrodontia of permanent central incisors, with a chief complaint of unerupted permanent central incisors, treatment was performed through the extraction of all supernumeraries, and interceptive partial fixed orthodontic treatment 2x4 appliance was used for nine months. The case will be treated with comprehensive orthodontics after the completion of the secondary dentition.

## Conclusions

Supernumerary teeth could present in the primary or secondary dentition, and their existence might be overlooked. The patient should be examined clinically and radiographically when supernumerary is suspected to establish a confirmed diagnosis. Furthermore, if a supernumerary tooth is reviewed, the location, crown direction, and resorption of adjacent roots must be scrutinized.

It is of great value to ensure early recognition of the existence of supernumeraries. On the contrary, if supernumerary teeth are left unattended, they may result in many complications, and the management will require an interdisciplinary approach to deliver the best treatment options.
